# Symphony of Success: Leader-Practitioner Reciprocity during Evidence-Based Practice Implementation

**DOI:** 10.1007/s10488-024-01405-y

**Published:** 2024-08-17

**Authors:** Karina Myhren Egeland, Marisa Sklar, Gregory A. Aarons, Mark G. Ehrhart, Ane-Marthe Solheim Skar, Randi Hovden Borge

**Affiliations:** 1 Norwegian Centre for Violence and Traumatic Stress Studies (NKVTS), Gullhaugveien 1, Oslo, 0484 Norway; 2 Department of Psychiatry, University of California, 9500 Gilman Drive (0812), La Jolla, San Diego, CA 92093-0812 USA; 3 UC San Diego ACTRI Dissemination and Implementation Science Center, 9500 Gilman Drive, La Jolla, CA 92093 USA; 4 Child and Adolescent Services Research Center, 3665 Kearny Villa Rd., Suite 200N, San Diego, CA 92123 USA; 5 Department of Psychology, University of Central Florida, 4111 Pictor Lane, Orlando, FL 32816-1390 USA; 6 National Institute of Occupational Health, Gydas vei 8, Oslo, 0363 Norway

**Keywords:** Followership, Implementation citizenship behavior, Implementation leadership, Reciprocal relationships, Social exchange hypothesis

## Abstract

This study aimed to explore the reciprocal relationships between implementation leadership and practitioner implementation citizenship behavior during the implementation of evidence-based practices (EBPs). Data were collected at two timepoints with a time lag of six months during a national implementation of evidence-based treatment for post-traumatic stress disorder in Norwegian mental health clinics. Data from 72 leaders and 346 practitioners were analyzed with a two-wave cross-lagged panel model, accounting for the nested structure and adjusting for demographic variables. Significant positive autoregressive effects for both implementation leadership and implementation citizenship behavior indicated some stability in ratings across time. Significant cross-lagged effects in both directions indicated that practitioners who experienced greater implementation leadership from their leaders demonstrated greater implementation citizenship behavior six months later, and vice versa. Findings hence supported both the social exchange hypothesis and the followership hypothesis, suggesting reciprocal associations between the constructs. The findings underscore the mutually influential relationship between leaders’ behavior and practitioners’ engagement in citizenship behavior during EBP implementation. The study emphasizes the importance of interventions focusing on leadership behaviors that encourage practitioner engagement and mutually beneficial behavior patterns, highlighting the reciprocal and vital roles that both leaders and practitioners play in successful EBP implementation.

## Background

For organizations to succeed with evidence-based practice (EBP) implementation, it is essential for the organizational members to actively engage and put extra effort into the implementation process (Damschroder et al., 2009). While leaders’ role in promoting practitioners’ implementation efforts has been examined in previous research (e.g., Aarons et al., 2015; Williams et al., 2022), the contribution of practitioners themselves in the leadership process is often overlooked, contributing to the perception that practitioners are passive recipients of leadership. Yet, according to followership theory and research (Oc et al., 2023; Uhl-Bien et al., 2014), followers’ relationships with leaders are based on mutual influence, and followers may have or take active roles in the leadership process. For implementation science and practice, this perspective may shed additional light on the critical roles of both leaders *and* practitioners in the implementation process, potentially providing the basis for changing clinical practice not only by intervening with leaders, but also with followers.

Implementation citizenship behavior is a type of behavior that practitioners perform that goes above and beyond basic job requirements in supporting the implementation of new practices (Ehrhart et al., 2015). Implementation citizenship behavior builds on the theories of more generic organizational citizenship behavior (OCB), defined as discretionary individual behavior that promotes the effective functioning of the organization (Organ et al., 2005). OCB has been widely studied since the 1980s (Organ, 2018), and meta-analyses have confirmed its links to a variety of individual-level (e.g., job performance ratings, rewards, turnover, absenteeism) and unit-level (e.g., productivity, efficiency, customer satisfaction) outcomes (Podsakoff et al., 2009). Furthermore, a recent systematic review showed several positive outcomes of OCB in the public sector, including improved individual performance, knowledge sharing, and physical and mental health (de Geus et al., 2020).

Among a variety of potential contextual, attitudinal, and dispositional antecedents, leadership is typically viewed as one of the primary predictors of OCB (LePine et al., 2002; Organ & Ryan, 1995). Most of the OCB research builds on the assumption that the causal direction in this relationship is from leader behavior to employee OCB (Organ et al., 2005). This is in line with a social exchange approach, which is founded on the notion that followers engage in OCB to reciprocate good leader treatment (Cropanzano & Mitchell, 2005). Several studies have supported this hypothesis, showing positive associations between leadership and OCB (Ilies et al., 2007; Lee et al., 2020; Wang et al., 2011).

In contrast to this unidirectional perspective, Organ et al. (2005) argued that a bidirectional relationship between employee OCB and leader behavior may also be possible. This perspective is in line with followership theory (Uhl-Bien et al., 2014). In contrast to leader-centric approaches to leadership, which tend to view followers as passive participants “who dutifully carry out the orders, directives, and whims of the leader” (Shamir, 2007, p. 84), followership theories take a follower-centric approach to “reverse the lens” and recognize that followers may act and cause the leader to adjust or increase certain behaviors. Research from this perspective has examined how follower proactive behaviors influence both leader motivation (Carsten et al., 2018) and leader emergence (Jiang et al., 2021), as well as how upward influence tactics impact leader decision-making (Higgins et al., 2003; Yukl & Falbe, 1990). To date, implementation research studies from a followership perspective are limited.

Both leadership and citizenship behavior are potentially important constructs for implementation effectiveness, in line with implementation frameworks such as the Exploration, Preparation, Implementation, & Sustainment (EPIS) framework that highlights, among other things, leadership and individual practitioner characteristics within an organization’s inner context (Moullin et al., 2019). Leadership behaviors specifically focused on the goal of implementation effectiveness are captured by the construct of implementation leadership, which includes whether leaders are proactive, knowledgeable, supportive, and perseverant during EBP implementation (Aarons et al., 2014b). Several studies have been conducted linking implementation leadership and implementation outcomes (e.g., Egeland et al., 2023; Williams et al., 2022; Williams et al., 2020). In contrast, as a newer construct in the implementation literature, relatively little research has been conducted on implementation citizenship behavior. It consists of two categories of citizenship behaviors assumed to relate to implementation success: keeping informed about the implementation (e.g., familiarizing oneself with new routines) and helping others (e.g., assisting peers in learning new skills) (Ehrhart et al., 2015). In one of the few studies connecting leadership and implementation citizenship behavior, Borge et al. (2021) showed that leader-reported implementation citizenship behavior was positively and significantly correlated with practitioner-reported implementation leadership and practitioners’ intentions to use EBPs, such that practitioners who rated their leader higher on implementation leadership received higher implementation citizenship behavior ratings from their leader and reported higher intentions to use EBPs.

From a social exchange perspective, it could be that when practitioners experience leaders who support and value their implementation efforts, they will respond by going above and beyond what is expected from them, in other words, performing more implementation citizenship behavior. However, from a followership perspective, it could also be that practitioners’ explicit interest in and motivation towards the implementation pushes leaders to pay more attention to what they can do to support followers’ efforts as well. Research to date cannot tell us whether leaders influence practitioners’ citizenship behavior or whether practitioners’ citizenship behavior makes leaders perform more leadership behavior. This is an important question to answer, not only to advance the scientific understanding of the directionality of relationships, but also to provide empirical support for an alternative pathway to initiate change during EBP implementation, namely that of intervening with follower behavior directly. Thus, we will investigate the following hypotheses using dyadic data consisting of leaders’ ratings of practitioners’ implementation citizenship behavior, and practitioners’ ratings of their leaders´ implementation leadership behavior:

### Hypothesis 1

Implementation leadership predicts implementation citizenship behavior six months later (i.e., social exchange hypothesis).

### Hypothesis 2

Implementation citizenship behavior predicts implementation leadership six months later (i.e., followership hypothesis).

## Methods

### Procedure

Data were collected during a national implementation of evidence-based treatment for post-traumatic stress disorder (PTSD) in Norwegian child and adult specialized mental health clinics (Egeland et al., 2019).

The implementation took place between August 2018 and June 2022. Invitations to participate in the implementation were sent to health trusts and clinic leaders. Interested clinics contacted the implementation team at the Norwegian Center for Violence and Traumatic Stress Studies (NKVTS) and received further information through e-mail, telephone, and face-to-face meetings. In the clinics that chose to participate in the implementation, a sub-sample of practitioners received training and supervision in one of three EBPs for PTSD (Trauma-Focused Cognitive Behavioral Therapy in child clinics; Cognitive Therapy for PTSD and Eye Movement Desensitization & Reprocessing in adult clinics) (National Institute for Health and Care Excellence, 2018). To support the implementation, the clinics participated in the Leadership and Organizational Change for Implementation (LOCI) strategy (Aarons et al., 2017, 2024; Egeland et al., 2019; Williams et al., 2023).

Data used in the current study were collected at two timepoints with a time lag of six months between measurements: (1) after the practitioners had received the EBP training and the leaders started engaging in the LOCI strategy, and (2) at the end of the LOCI strategy. Participation in the survey study was voluntary and based on informed consent. At both timepoints, the leaders completed surveys reporting on their practitioners’ implementation citizenship behavior and practitioners completed surveys reporting on their leader’s implementation leadership behaviors. Thus, the data had a dyadic structure, consisting of other-ratings (as opposed to self-ratings) on both implementation leadership and implementation citizenship behavior. The timing of both measurements ensured that the leaders and practitioners had enough time to observe each other’s implementation-related behaviors. Online surveys were administered via email to all participants. Reminders were sent to those who had not responded 14 and 28 days after the first invitation. The project was approved by the Norwegian Centre for Research Data (No. 60036/3/LH and 60059/3/OOS).

### Participants

Potential participants were 75 first-level leaders from the mental health clinics participating in the implementation project and 426 practitioners who had received training in the EBPs being implemented. The 70 participating mental health clinics were spread across all four regional health trusts (North, West, South-East, and Central) of Norway. Among the practitioners, 272 and 246 responded to questions about implementation leadership at T1 (64%) and T2 (58%), respectively. Among the leaders, 72 out of 75 responded to the survey at both T1 and T2 (response rate = 96%). The mean number of practitioners per leader was 4.06 at T1 (range 1–9) and 3.73 at T2 (range 1–8). The total sample of practitioners across timepoints was 346, and 174 responded at both T1 and T2. As shown in Table 1, the sample included a majority of women (79.5%) and had an average age of around 43 years (SD = 10.5). The average tenure in their current occupation was 11 years (SD = 9.0). The leader sample consisted entirely of first-level leaders with direct supervision over clinical practice, where a considerable part of the job involved interacting with the practitioners. The majority was women (65.3%) and the average age was 50 years (SD = 7.9). The average tenure as leader within the current organization was 13 years (SD = 9.9).

**Table 1:** Participant demographics (*N* = 346)

		Practitioners (*N* = 346)	Leaders (*N* = 72)
Gender			
Women Men Missing	*N* (%) *N* (%) *N* (%)	275 (79.5) 54 (15.6) 17 (4.9)	47 (65.3) 24 (33.3) 1 (1.4)
**Age** Missing	M (SD) N (%)	42.6 (10.5) 26 (7.5)	49.7 (7.9) 1 (1.4)
**Current work experience (years)** Missing	M (SD) N (%)	11.3 (9.0) 48 (13.9)	13.3 (9.9) 1 (1.4)
Education			
Medical doctor/psychiatrist Clinical psychologist Psychiatric nurse Other Missing	N (%) N (%) N (%) N (%) N (%)	41 (11.9) 182 (52.6) 46 (13.3) 53 (15.3) 24 (7.0)	7 (9.7) 32 (44.4) 16 (22.2) 16 (22.2) 1 (1.4)

### Measures

Implementation citizenship behavior was measured with the Implementation Citizenship Behavior Scale (ICBS) (Ehrhart et al., 2015). The scale consists of six items representing into two subscales: (1) *helping others* and (2) *keeping informed*. Based on data from the current study, the subscales (α = 0.95 and α = 0.92, respectively) and the total scale showed excellent internal consistency (α = 0.95) (Borge et al., 2021). As recommended by the scale developers, item language was tailored to the implementation of the EBP being implemented, in this case for PTSD (e.g., “Keeping informed of changes in policies and procedures regarding evidence-based practice for PTSD”).

Implementation leadership was measured with the Implementation Leadership Scale (ILS) measuring first-level leadership behaviors thought to impact EBP implementation (Aarons et al., 2014b). The scale consists of 12 items divided into four subscales: (1) *proactive* describes the degree to which the leader anticipates and addresses implementation challenges, (2) *knowledgeable* refers to the degree to which a leader has a deep understanding of EBP and implementation issues, (3) *supportive* measures the degree of the leader’s support of followers’ adoption and use of EBP, and (4) *perseverant* refers to the degree to which the leader is consistent, unwavering, and responsive to EBP implementation. Based on data from the current study, the subscales (α = 0.93 – α = 0.97) and the total scale (α = 0.96) showed excellent internal consistency (Braathu et al., 2022). As recommended by the scale developers, item language was tailored to the implementation of EBP for PTSD (e.g., “[The leader] is knowledgeable about evidence-based practice for PTSD”).

### Statistical analyses

Study hypotheses were investigated with a two-wave cross-lagged panel model (CLPM) in Mplus 8.3 (Muthén & Muthén, 2017) adjusting for the nested data structure (TYPE = COMPLEX) and using full information maximum likelihood estimation with robust standard errors (MLR). An unconstrained CLPM estimates all autoregressive and cross-lagged relationships between the study variables simultaneously. The autoregressive parameter represents the degree to which an individual’s observed level on one variable on the first timepoint predicts their observed level on the same variable on the second timepoint (e.g., do practitioners who rated their leader high on ILS on the first timepoint tend to do so also on the second timepoint? ). The cross-lagged parameter represents the degree to which an individual’s observed level of one variable on the first timepoint predicts their observed level on a different variable on the second timepoint (e.g., do practitioners who rated their leader high on ILS on the first timepoint tend to receive high ratings of implementation citizenship behavior on the second timepoint? ). Thus, the CLPM captures the degree to which one variable predicts the other variable at the subsequent timepoint, above and beyond prior levels of the latter (Orth et al., 2022). The lagged relationships were also adjusted for age (in years), sex (male or female), work experience (in years), and the implementation period (indicated by two dummy variables for the second and third period). Implementation citizenship behavior and implementation leadership were included as manifest variables based on mean scores calculated by averaging across items of the ICBS and ILS, respectively.

## Results

**Fig. 1:**
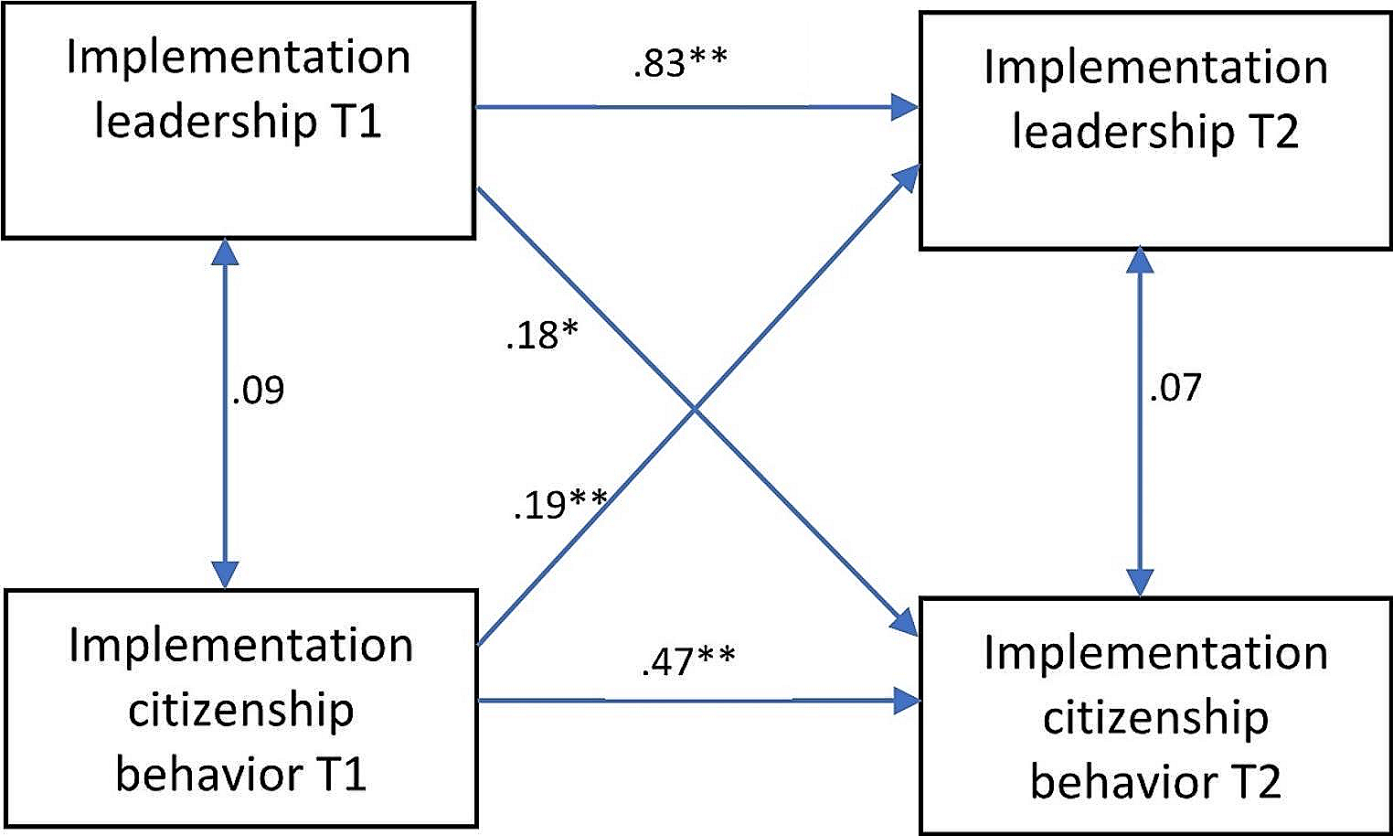
Unconstrained cross-lagged panel model; unstandardized coefficients; *p < .05; **p < .01

The cross-lagged panel model showed significant and positive autoregressive effects for both implementation leadership and citizenship behavior (Fig. 1; Table 2). This indicates that practitioners who, relative to others, rated their leaders high on timepoint 1 tended to also rate their leaders high on timepoint 2. Similarly, practitioners rated high on implementation citizenship behavior from their leaders on timepoint 1, were also rated high on implementation citizenship behavior on timepoint 2. The autoregressive effect was significantly higher for implementation leadership (*b* = 0.83) than for implementation citizenship behavior (*b* = 0.47) (*W* (1) = 7.96, *p* = .01), indicating that implementation leadership ratings tended to be more stable over time than implementation citizenship behavior ratings.

**Table 2:** Model results

	Unstandardized		Standardized	
	Estimate	S.E.	Estimate	S.E.
IL timepoint 1 - ICB timepoint 2	0.18*	0.09	0.20*	0.09
IL timepoint 1 - IL timepoint 2	0.83**	0.07	0.73**	0.04
ICB timepoint 1 - ICB timepoint 2	0.47**	0.10	0.40**	0.08
ICB timepoint 1 - IL timepoint 2	0.19**	0.07	0.13**	0.05
R^2^ ILS timepoint 2	0.58**	0.05		
R^2^ ICBS timepoint 2	0.33**	0.07		

Note. IL = implementation leadership; ICB = implementation citizenship behavior

* p = < 0.05; ** p = < 0.01

The model showed statistically significant and positive cross-lagged effects of both implementation leadership and implementation citizenship behavior (Fig. 1; Table 2). This indicated that practitioners who rated their leader high on implementation leadership on timepoint 1 tended to be rated high on citizenship behavior by their leader on timepoint 2. Furthermore, practitioners who were rated high on citizenship behavior on timepoint 1, tended to rate their leader high on implementation leadership on timepoint 2. The two cross-lagged effects were not significantly different from each other (*W* (1) = 0.00, *p* = .96). Based on effect size guidelines recently suggested by Orth et al. (2022), both cross-lagged effects can be categorized as large (i.e., standardized effects above 0.12). These results are in line with both the social exchange hypothesis and the followership hypothesis, indicating reciprocal associations between the two constructs over time. The association between the variables at T1 and between the error variances at T2 were both statistically non-significant. A comparison of model fit across models with differing constraints corroborated the above results; the unconstrained model provided the best fit to the data, whereas the model with both cross-lagged effects constrained to zero fit the data significantly worse, as did models constraining only one of the two cross-lagged effects to zero (Table 3).

**Table 3:** Model Fit results

Model	χ^2^ (df) (*p*-value)	RMSEA (*p* < .05)	CFI
Model 1: Unconstrained (saturated) model	χ^2^(0) = 0.00 (0.000)	0.00 (0.00)	1.00
Model 2: CL path from IL to ICB constrained to zero	χ^2^(1) = 6.59 (0.010)	0.13 (0.05)	0.96
Model 3: CL path from ICB to IL constrained to zero	χ^2^(1) = 6.36 (0.001)	0.12 (0.06)	0.96
Model 4: Both CL paths constrained to zero	χ^2^(2) = 12.96 (0.002)	0.13 (0.02)	0.93

Note. CFI = comparative fit index; RMSEA = root mean square error of approximation; CL = cross-lagged; IL = implementation leadership; ICB = implementation citizenship behavior

### Additional Analyses

Since implementation citizenship behavior is a relatively new and understudied construct, we also investigated whether there were significant differences between the cross-lagged effects of implementation leadership on the implementation citizenship behavior subscales *helping others* and *keeping informed*. A Wald test of model constraints indicated that the two cross-lagged effects were not significantly different from each other (*W* (1) = 0.09, *p* = .77), which suggests that associations between implementation citizenship behavior and implementation leadership are consistent whether considering the ICBS total scale or its individual dimensions.

## Discussion

The current study is the first of its kind to investigate reciprocal relationships between leaders’ implementation leadership and practitioners’ implementation citizenship behavior. Our findings indicated that practitioners who reported more implementation leadership behaviors from their leader exhibited more implementation citizenship behavior six months later, as reported by their leader. This is in line with our hypothesis based on social exchange theory (Cropanzano & Mitchell, 2005), positing that leaders, through their implementation leadership behavior, may promote practitioners’ citizenship behavior during EBP implementation.

Our findings further indicated that practitioners who exhibited more implementation citizenship behavior reported more implementation leadership behaviors from their leader six months later. This is in line with our followership hypothesis, where we expected based on the “reversing the lens” approach to leader-follower relationships (Shamir, 2007; Uhl-Bien et al., 2014) that leaders might engage in more implementation leadership towards practitioners who go above and beyond to promote and support EBP implementation.

Our findings of significant cross-lagged effects in both directions and of similar magnitude suggest reciprocal relationships between implementation leadership and implementation citizenship behavior. This is in line with followership approaches to leadership (Oc et al., 2023; Uhl-Bien et al., 2014), and suggests that to fully understand leadership in the context of EBP implementation, it is essential to consider the role of practitioners not only as recipients of implementation leadership, but also as potential drivers of leader behavior. This finding could be interpreted to mean that practitioners demonstrating high implementation citizenship behavior receive more support from their leaders, which is critical because informal roles as change agents likely require additional support and rewards to reinforce them in these efforts. Our findings further hint to positive feedback loops at the practitioner level; more implementation leadership may foster more citizenship behavior, which may again foster more implementation leadership. This calls for future research that includes more measurement occasions to examine potential feedback loops over time.

Yet, our findings that practitioners who exhibited more citizenship behavior reported more implementation leadership behavior from their leader later on, does not only have positive implications, but also implies that practitioners who exhibited less citizenship behavior on timepoint 1 gave lower ratings of implementation leadership on timepoint 2. In other words, our findings suggest the possibility that practitioners who might have needed it the most (e.g., low levels of citizenship behavior may signal a lack of motivation) received less attention relative to their peers. Increased awareness of this potential pitfall can help leaders direct more attention to practitioners who need extra support. Our findings underscore that there may be important benefits associated with measuring and monitoring citizenship behavior with tools such as the ICBS (Ehrhart et al., 2015), in order to proactively and effectively support both practitioners high in citizenship, thus taking on informal leadership roles, and those who are less engaged and may also need more support. Furthermore, while leadership represents one important source of such support, it is not the only one. The implementation process is complex, and a combination of interventions and support from different organizational levels may be necessary to sustain practitioner engagement over time and to succeed with EBP implementation (Moullin et al., 2019).

Compared to other constructs at the practitioner level such as EBP attitudes (Aarons, 2004) or implementation intentions (Moullin et al., 2018), implementation citizenship behavior has received far less attention. A distinct aspect of implementation citizenship behavior is the strong emphasis on practitioners’ role as change agents in EBP implementation (Lindner et al., 2020). This highlights its dual nature as both a desirable outcome in EBP implementation and as a potential cause of change. Thus, future studies should focus not only on what influences implementation citizenship behavior and EBP use (Gurses et al., 2010), but also on how implementation citizenship behavior may be reciprocally related to a variety of factors at both the individual and organizational level, such as implementation climate (Aarons et al., 2014b), or organizational culture more broadly.

### Strengths and Limitations

This study stands out from other studies that are based on self-report data measured at the same timepoint and from the same source (Organ, 2018), as it incorporates data measured at two timepoints from two different sources rating each other. This design enabled us to examine how the level of one variable predicted the level of another above and beyond initial levels of the latter, as well as bypassing challenges related to common method bias. Thus, the study is better equipped to approximate a potential causal effect than previous studies of similar relationships (Haider et al., 2017; Nohe & Hertel, 2017).

Yet, it is important to note that although our findings are in line with our hypotheses, causal interpretations should be made cautiously. There are many forces driving both leadership (Yukl & Gardner, 2020) and citizenship behaviors (Organ et al., 2005). Thus, there may be common causes of both constructs that we did not account for through our study design and analyses. These include organizational setting characteristics driving both behaviors, such as financial and non-financial resources that may facilitate the engagement in both implementation leadership and implementation citizenship behavior, or dispositional and attitudinal characteristics of individuals. One alternative explanation related to the latter could be that practitioners who were more positive towards the EBP being implemented exhibited more implementation citizenship behavior, and that this positive view also colored their perception of the leader’s implementation leadership. One way for future studies to address these concerns is to include more measurement occasions to allow for a decomposition into stable individual differences between persons and time-varying fluctuations at the within-person level (Hamaker et al., 2015). Additionally, the current study comprises a limited dataset wherein responses from both practitioners and leaders were required to explore the associations, potentially posing challenges to external validity.

The data were collected in the context of a larger implementation trial. This may have biased observed relationships and may also challenge the generalizability of findings. In terms of bias, there may be mechanisms operating in opposite directions. On one hand, participants might have been more attentive and observant of each other’s behavior than they typically would be, which could have led to an *over*estimation of the strength of relationships. On the other hand, as part of the LOCI strategy, leaders received feedback on practitioners’ ratings of implementation leadership, which likely led to changes in subsequent leader behavior, particularly for those who scored below expectations on the first timepoint. This implies that the LOCI intervention may have led to a positive shift in all leaders’ behavior over time (as should be expected from a leadership intervention) and possibly also reduced variance in leadership ratings between leaders compared to how these processes would naturally occur without intervention. Thus, an *under*estimation of relationships also seems plausible. It is important to note, however, that feedback was given on unit-level data only (i.e., individual practitioners were not identified), and neither leaders nor practitioners received feedback on citizenship behavior. The stability in the constructs was also fairly high, particularly for implementation leadership. The fact that implementation citizenship behavior predicted implementation leadership above and beyond this stability strengthens evidence for the reciprocal nature of the relationship between the two constructs.

### Practical Implications

In line with previous studies linking leadership behavior and OCB in general (Ilies et al., 2007; Lee et al., 2020; Wang et al., 2011), our findings suggest that leaders may play an important role in promoting implementation citizenship behavior among practitioners by demonstrating strong implementation leadership. Organizations should consider interventions focused on enhancing leadership behaviors that encourage and support practitioners to perform above and beyond basic job requirements during EBP implementation. Leaders should, however, also be mindful of practitioners who exhibit less implementation citizenship behavior, as they might require additional supportive resources provided by the leaders themselves, peers, or from higher levels in the organization.

Acknowledging the bidirectional nature of the relationship between implementation leadership and citizenship behavior is pivotal. There may be natural “champions” of the use of evidence-based practice that can enhance leader initiatives toward improved practice (Santos et al., 2022). Current perspectives within implementation science and practice view leaders as change agents and providers of guidance (Meza et al., 2021), but our findings suggest that we should also recognize *practitioners* as potential contributors to leadership dynamics during EBP implementation. Thus, while implementation strategies often focus on intervening with leaders to change follower behavior, intervening with followers may provide an additional pathway for bringing about change. Importantly, effective unit-level strategies or interventions to enhance and sustain citizenship behavior may not only involve leadership support. Drawing on the general OCB literature, alternative strategies may include, for instance, increasing team identification and peer support (Chiaburu & Harrison, 2008; Farmer et al., 2015) or the reframing of work roles to include certain citizenship behaviors (Parke et al., 2021).

## Conclusion

This study provides valuable insights into the reciprocal relationship between leaders’ implementation leadership and practitioners’ implementation citizenship behavior within EBP implementation. On one hand, the results suggest that effective implementation leadership may promote practitioners’ later engagement in implementation citizenship behavior. On the other hand, practitioners who exhibited greater implementation citizenship behavior reported higher implementation leadership from their leader later in the implementation process. These findings hint to a dynamic and mutually influential relationship between leaders and practitioners in driving EBP implementation success, although it is important to highlight that alternative explanations could explain the observed patterns. When engaging in implementation leadership behaviors, leaders should be cognizant of practitioners with low levels of implementation citizenship behavior and consider amplifying implementation leadership behaviors toward these practitioners to ensure a positive implementation climate. Future interventions may choose to focus on nurturing leadership behaviors that encourage practitioner engagement in citizenship behaviors during EBP implementation, thus fostering a potentially reciprocal relationship between leaders and practitioners in driving successful implementation efforts.
