# Correction to: Symphony of Success: Leader-Practitioner Reciprocity during Evidence-Based Practice Implementation

**DOI:** 10.1007/s10488-024-01414-x

**Published:** 2024-09-26

**Authors:** Karina Myhren Egeland, Marisa Sklar, Gregory A. Aarons, Mark G. Ehrhart, Ane-Marthe Solheim Skar, Randi Hovden Borge

**Affiliations:** 1 Norwegian Centre for Violence and Traumatic Stress Studies (NKVTS), Gullhaugveien 1, Oslo, 0484 Norway; 2 Department of Psychiatry, University of California, 9500 Gilman Drive (0812), La Jolla, San Diego, CA 92093-0812 USA; 3 UC San Diego ACTRI Dissemination and Implementation Science Center, 9500 Gilman Drive, La Jolla, CA 92093 USA; 4 Child and Adolescent Services Research Center, 3665 Kearny Villa Rd., Suite 200N, San Diego, CA 92123 USA; 5 Department of Psychology, University of Central Florida, 4111 Pictor Lane, Orlando, FL 32816-1390 USA; 6 National Institute of Occupational Health, Gydas vei 8, Oslo, 0363 Norway

**Correction to: Administration and Policy in Mental Health and Mental Health Services Research**

10.1007/s10488-024-01405-y

In the original version of this article, the given and family names of all authors were incorrectly structured.

The author group has been updated above and the original article has been corrected.

